# Evaluation of immunoglobulin-Y in place of tylosin phosphate in the diets fed to Holstein Steers and preliminary analysis of liver abscess duration on animal growth performance

**DOI:** 10.1093/tas/txaa225

**Published:** 2020-12-05

**Authors:** Miranda K Stotz, Darren D Henry, Whitney L Crossland

**Affiliations:** Department of Animal and Food Sciences, Texas Tech University, Lubbock, TX

**Keywords:** antibiotic alternative, Holstein, immunoglobulin, liver abscess, ultrasound

## Abstract

Despite the regular use of feed-grade macrolide-antibiotics, bovine liver abscesses persist, representing a financial burden to pre- and post-mortem sectors of the beef industry. An immunoglobulin-Y (**IGY**) additive developed to target *Fusobacterium necrophorum* and *Trueperella pyogenes,* was evaluated for the control of liver abscesses. Research is needed for the impact of liver abscess severity as well as abscess duration on steer performance and carcass characteristics. Holstein steers (*n* = 64; initial body weight (**BW**) = 372.5 ± 2.41 kg) consuming a finishing diet for 188 d were used in a completely randomized design where treatments included: **TYL** (tylosin phosphate 90 mg/d; *n* = 32) or IGY (2.5 g/d; *n* = 32) and steer was the experimental unit. Feed intake was recorded daily while BW and liver ultrasound outcome (normal or abnormal) was recorded every 28 d until slaughter to estimate duration of abscess presence (**DUR**). Continuous variables of animal growth performance and carcass characteristics were analyzed using the MIXED procedure of SAS. Categorical quality grade and liver data were analyzed using the GLIMMIX procedure of SAS. Treatment did not affect live or carcass-adjusted growth performance (*P ≥* 0.131). However, steers fed TYL had greater (*P* = 0.042) empty body fat (**EBF**) % and a greater proportion of carcasses grading premium choice than steers fed IGY (*P* = 0.030). Treatment did not affect prevalence of abscessed livers, abscess severity or estimated abscess duration (*P ≥* 0.213) but datasets with greater experimental units are needed to substantiate this outcome. Increasing abscess severity tended (*P* ≤ 0.10) to linearly reduce carcass-adjusted gain to feed (**G:F**), fat thickness, and EBF. Carcass dressing % was only affected by severe (A+ and A+AD) abscess scores (*P* = 0.010). Carcass-adjusted final BW, average daily gain, G:F, and hot carcass weight was decreased only when the estimated DUR was *≥*140 d (*P* ≤ 0.05). Carcass dressing %, however, was linearly affected by estimated liver abscess DUR (*P* ≤ 0.005), regardless of abscess severity. Preliminary evidence suggests that measuring the duration of liver abscess affliction during the feeding period may also give insight to the degree of performance reduction.

## INTRODUCTION

Liver abscesses in finishing cattle continue to reduce beef production efficiency for both cattle feeders and packers, regardless of feed-grade antibiotic use for their control (Nagaraja and Chengappa, 1998; McKeith et al., 2012). The liver may account for 20–25% of the oxygen consumption by cattle (Huntington and Reynolds, 1987; Eisemann et al., 1996), highlighting its relative importance in overall metabolism, and when damaged, effect on metabolic efficiency. Over the last 30 years, the mean proportion of liver abscesses among feedlot cattle at harvest has ranged from 19.2% to 30.8% (Eastwood et al, 2017) and may be influenced by factors such as sex, days on feed, physically effective fiber in the diet, bunk management, and dairy cattle influence (Brink et al., 1990; Nagaraja and Chengappa, 1998; Nagaraja and Lechtenberg, 2007). Numerous articles have drawn attention to the negative effects of liver abscesses on finishing cattle growth performance and carcass value (Brink et al., 1990; Nagaraja and Chengappa, 1998; Nagaraja and Lechtenberg, 2007; Brown and Lawrence, 2010). Holstein steers currently represent approximately 20% of the nationwide fed-beef population and generally experience greater incidence and severity of liver abscesses than beef cattle (Amachawadi and Nagaraja 2016). However, there have been no studies which report the comparative impact of liver abscess severity on Holstein steers and no reports which estimate the length of time cattle are affected by abscesses in the finishing period. We hypothesized that duration of abscess burden in the feedlot, regardless of severity, may also impact finishing cattle growth performance and carcass characteristics.

Bovine liver abscesses commonly involve *Fusobacterium necrophorum* and *Trueperella pyogenes* as etiological agents (Lechtenberg et al., 1988; Tan et al., 1996). Tylosin phosphate is a broad-spectrum antibiotic fed to approximately 57% of feedlot cattle (USDA, 2019) to prevent, control, or treat liver abscess infections with an abscess reduction rate of 40–70% (Nagaraja and Lechtenberg, 2007; Depenbusch et al., 2008; Reinhardt and Hubbert, 2015). However, multiple trials have concluded that the use of tylosin phosphate significantly increases the proportion of antimicrobial resistant bacteria shed in the feces of cattle (Jacob et al., 2008; Zaheer et al., 2013; Beukers et al., 2015). The greater awareness of the dissemination of resistant genes in the food animal industry sector has led to research efforts to identify feed-grade antibiotic alternatives. One such alternative is immunoglobulin-Y, which mainly plays a role in humoral immunity of egg-laying species (Larsson and SjöQuist, 1990; Polanowski et al., 2012). The immunoglobulin can be harvested from the egg yolk of hens that have been hyper-immunized using pre-determined antigens. Due to its high specificity, it was hypothesized that the use of immunoglobulin-Y to target bacteria of concern could serve as a viable antibiotic alternative in prevention of hepatic abscesses in Holstein finishing steers.

Therefore, the two main objectives of this study were to (1) evaluate the efficacy of feeding a customized immunoglobulin-Y complex, developed to specifically target *F. necrophorum* and *T. pyogenes*, in place of tylosin phosphate for the control of liver abscesses, and (2) conduct a preliminary investigation of the effects of the estimated liver abscess duration on feeding performance and carcass characteristics of Holstein steers to be considered among the established literature of comparative liver abscess research.

## MATERIALS AND METHODS

Subjects were cared for under a protocol approved by the Texas Tech University Institutional Animal Care and Use Committee (#18080-10).

### Experimental Design and Subjects

This experiment was a completely randomized design with two dietary treatments. The trial was conducted for a duration of 188 d with individual steers serving as the experimental unit. Seventy-seven Holstein steers (delivered shrunk body weight (**BW**) = 301.64 ± 8.45 kg) were locally sourced from a commercial feedlot in the high plains of Texas and transported 217 km to the Texas Tech University Beef Center located 9.7 km east of New Deal, TX. The cattle source had a historic liver abscess prevalence of approximately 60% for Holstein type steers consuming a finishing diet including tylosin phosphate (90 mg/d) for the control of liver abscesses. Steers were previously adapted to a high concentrate diet prior to transport to the experimental location and fed tylosin phosphate for approximately 140 d prior to arrival. Upon arrival (d −52), steers were tagged with a unique identification number via radio frequency identification tag (Allflex USA, Inc., Dallas, TX). Body weight was recorded using a Digi-Star scale system which was calibrated before each weigh session (Digi-Star LLC. Fort Atkinson, WI). Steers were fed a high-concentrate finishing diet, with similar ingredient, energy, and protein composition specifications to the commercial feedlot diet at their previous location, in common groups for 14 d in concrete bunks. On d −37, steers were examined for the presence of preexisting liver abnormalities using external abdominal ultrasonography (Hitachi Aloka 500, 3.5 MHz curvilinear probe; Tokyo, JPN) by a veterinarian (Dr. Eila Machado, DVM). Liver images were captured from the intercostal spaces between ribs 9 and 13 and live video feed was analyzed from ultrasound screen to evaluate normal or abnormal hepatic tissue. Normal or abnormal hepatic tissue was determined based on research by Braun (2009) and Lechtenberg and Nagaraja (1991) which discuss the normal liver parenchyma and disease state presentation using ultra-sonographic imaging. Steers with obvious abnormal liver tissue (*n* = 2 steers; presumed abscess) were excluded from trial participation so as not to confound previous liver condition within treatment. Remaining steers which were greater than two standard deviations (±) of the mean BW were excluded from the trial. Sixty-four steers were then stratified by BW (372 ± 12 kg) across 2 pens (*n* = 32/pen) each equipped with an automated feed intake monitoring system (SmartFeed bunks, C-Lock Inc., Rapid City, SD; 4 bunks/pen). Steers were monitored daily for feeding irregularities and general health and were considered adapted to the feeding system once the accountable feed balance was >96% for 14 consecutive days. During the adaptation period, six steers were removed from the trial for failure to adapt to the feeding system, one was removed later due to illness, and another was removed for injury (final *n* = 56).

### Diets, Treatments, and Feeding

Upon arrival, steers were fed a high concentrate diet ad libitum (at 10% refusal rate; Table 1). The diet was formulated using the Beef Cattle Nutrient Requirements Model (NASEM, 2016) where the carrier for feed additive treatments was ground corn included at 2% (DM basis). The positive control was carrier plus tylosin phosphate (**TYL**; Tylan® Premix, Elanco Animal Health, Greenfield, IN) delivered at the maximum labeled rate of 90 mg/day (actual intake 95% confidence interval was 90 ± 14.5 mg/d) and was fed to all cattle until d 0. On d 0, one pen of steers remained on the established dietary treatment, TYL (*n* = 27), while the other pen of steers began the new feed treatment containing immunoglobulin-Y and no tylosin phosphate (*n* = 29).

**Table 1.** Ingredient and chemical composition of basal diet fed to Holstein steers without the inclusion of TYL^*^ or IGY^*^

*Item* ^*^	*Diet*
% of diet, DM	
Steam-flaked Corn	63.0
Corn-gluten feed	23.5
Cotton Burrs	6.00
Ground Corn^†^	2.00
Tallow	1.20
Urea	0.30
Limestone	2.00
Supplement^‡^	2.00
Dry matter, % of diet	76.9
Crude protein, %	13.4
Acid detergent fiber, %	10.5
Crude fat, %	4.57
Calcium, %	0.82
Phosphorus, %	0.48
Total starch, %	51.0
NEm, Mcal/kg^§^	2.16 (2.12)
NEg, Mcal/kg^§^	1.48 (1.45)

^*^Items are feed ingredients and chemical composition of diets (dry matter basis) evaluated by ServiTech Laboratories, Amarillo, TX.

^†^Carrier: inclusion rate of the diet set at 2%. Inclusion rate of treatment (tylosin phosphate (90 mg/d) or immunoglobulin-Y (2.5 g/d) was based on steers per pen and 10% allowable refusals.

^‡^Supplement concentration on DM basis includes 2.5% potassium, 0.02% magnesium, 0.26% sulfur,17.5% salt, 6.93% sodium, 12.98% chlorine, 10 mg/kg cobalt, 500 mg/kg copper, 280.12 mg/kg iron, 25 mg/kg iodine, 1,500 mg/kg manganese, 2.5 mg/kg selenium, 4,000 mg/kg zinc, 110,000 IU/kg Vit. A, 17.5 IU/kg, 875 IU/kg Vit. E, and 1,500 g/ton ionophore (Rumensin-90, Elanco Greenfield, IN).

^§^Tabular values of dietary NEm = Net energy allowable for maintenance and NEg = Net energy allowable for gain. Parenthesis are values calculated from performance where: NEm = 2.12 Mcal/kg, and NEg = 1.45 Mcal/kg (NASEM, 2016).

The immunoglobulin-Y product was developed and harvested by Camas, Inc. (Le Center, MN) as an antibody product from laying hens hyper-immunized with *F. necrophorum* spp. *necrophorum* and *T. pyogenes* (**IGY**_**F+T**_), using two wild-type isolates obtained from the College of Veterinary Medicine at Kansas State University, and recommended by the manufacturer to be fed at a rate of 2.5 g/d in the diet (actual intake 95% confidence interval was 2.5 ± 0.41 g/d). Feeding rate of IGY_F+T_ was based on previous research by DiLorenzo et al. (2008) who fed a similar product egg product. Samples of IGY_F+T_ were collected randomly 3 times per month and sent to Camas, Inc. for quality of titer analysis. The IGY_F+T_ was mixed with ground corn carrier before each feeding event (the carrier and IGY_F+T_ = **IGY**). Both treatments were fed at the recommended dose, plus 10% to account for ad libitum delivery of feed, while carrier was scaled to projected intake based on previous weeks feed residuals.

Steers were fed twice daily at 0600 and 1700 hours. Treatment was mixed with carrier using a hand mixer, for each respective premix, to deliver the desired per diem amount based on the dry matter intake of each treatment group the previous week. Then, the premix was added directly to a Roto-Mix trailer (model 84–8, Dodge City, KS) at the base of the power take-off shaft in order to ensure best distribution throughout each batch. To prevent treatment contamination, a treatment-free batch of diet was supplied to the Roto-Mix trailer and delivered to off-trial steers in between delivery of treatment diets. Feed bunk refusals were cleared weekly and after major precipitation events. Diet samples were collected daily at 1700 hours and composited by week for dry matter (**DM**) determination, and by month for chemical analyses (ServiTech laboratories, Amarillo, TX; Table 1). Chemical analysis was conducted for crude fat (AOAC 920.39), acid detergent fiber (**ADF**; ANKOM Method 5 for A200), crude protein (AOAC 990.03), calcium and phosphorus (AOAC 990.08), and starch (AOAC 996.11). Net energy for maintenance and gain were tabulated based on diet composition and also back calculated based on steer performance (NASEM, 2016). Diet samples were dried at 100**°**C for 24 hours in a forced-air oven (Grieve, Round Lake, IL) for DM determination.

### Data Collection and Calculations

Finishing cattle growth performance and Hepatic Ultrasonography. Data from SmartFeed bunks were transferred via wireless internet transmission and downloaded to the online platform configured for the SmartFeed system. Individual as-fed intake was collected from the SmartFeed system and for the use of steer as an experimental unit. As-fed feed intake was converted to DM based on weekly DM values with fasting and weigh dates removed from the dataset. Mean daily DM intake (**DMI**) was calculated for d 0–d 188, d 0–d 84, and d 85–d188. Shrunk BW was obtained by withholding feed for 16–18 hours prior to BW measurements and collected on d 0, 28, 56, 84, 112, 140, 168, and 188 between 0600 and 1200 hours along with liver ultrasound imaging. Livers were visualized using the same method mentioned previously for the pre-trial screening. Live video was used to evaluate liver tissue for confluence and still images were saved for chronological visualization. Data were binomial outcomes of normal or abnormal tissue detected on each day of data collection and used for the estimation of abnormal tissue duration. Average daily gain (**ADG**) was calculated by subtracting the final BW from the initial BW divided by the days of feed. Interim ADG was also calculated for d 0–d 84 and d 85–d 188. Final shrunk BW was obtained on d 188 before shipment for harvest. The gain-to-feed ratio (**G:F**) was calculated by dividing ADG by DMI for the total days on feed and the intervals previously mentioned.

Carcass measurements, adjusted performance, and liver evaluation. Cattle were harvested at a commercial processing facility in TX. Personnel trained at Texas Tech University collected carcass data including: hot carcass weight (**HCW**), 12th rib subcutaneous fat thickness (**FT**), longissimus dorsi muscle area (**LMA**), and marbling score (**MARB**). Percent kidney, pelvic, and heart fat was not quantified due to plant procedures and therefore was assumed 3% of carcass fat for all carcasses. Yield grade was calculated using the USDA regression equation (USDA, 2017). Dressing percentage (**DP**) was calculated by dividing the HCW by the final shrunk BW taken on d 188. All livers were graded on a modified Elanco scale (Brown et al., 1975; Elanco, Greenfield, IN). All livers were removed from the harvest line for thorough examination to receive one of the following designations based on liver appearance: **0**, no abscesses or scars; **S**, resolved abscess scar; **A−**, one or two small abscess; **A**, one or two large abscesses or several small abscesses; **A+**, multiple large abscesses; **A+AD**, adhesion. One evaluator with no knowledge of dietary treatment graded all livers while an associate captured images of the liver (abdominal side).

Carcass-adjusted BW was calculated by dividing the HCW by the average DP of all steers on trial (61.8%). Carcass-adjusted ADG was calculated by subtracting the carcass-adjusted final shrunk BW from the initial shrunk BW and divided by 188 days on feed. Carcass adjusted G:F was calculated as carcass adjusted ADG divided by the overall mean DMI. Calculations for empty body fat (**EBF**) and adjusted final shrunk body weight (**AFBW**) were calculated according to equations summarized in Guiroy et al. (2001) using the common EBF of 28% for cattle grading low choice.

### Statistical Analysis

Individual steer was the experimental unit for this study. Continuous variables were analyzed as a completely randomized design using the MIXED procedure of SAS (9.4, SAS Institute Inc., Cary, NC) and the following model:

$$\begin{array}{l} Y=\mu +treatment+e. \end{array}$$

where *Y* represents response variables (initial BW, final BW, DMI, ADG, G:F, HCW, DP, FT, LMA, YG, MARB, carcass-adjusted final BW, carcass-adjusted ADG, carcass-adjusted G:F, EBF, AFBW), μ was the overall mean, treatment was the fixed effect of dietary treatment (TYL or IGY), and e represents random error associated with the measurement of the treatment. Three steers were removed from finishing cattle growth performance data analysis due to being extreme outlying observations for voluntary intake (final analyzed *n* = 53). Least squared means of DMI, ADG, and G:F were computed for d 0–84, 85–188, and d 0–188. Treatment effect on categorical quality grade and liver abscess prevalence, and severity were analyzed as binomial proportions using the GLIMMIX procedure of SAS with the ILINK option.

A post hoc analysis was performed to evaluate the outcome of liver severity, or the estimated liver abnormality (presumed abscess) burden duration (**DUR**), on live performance and carcass characteristics using the same statistical model used to assess treatment except that the abscess severity and DUR were used as fixed effects. Due to irregularly distributed outcomes of liver severity scores, scores were summarized into four categories of ordinal sequence 0, S, **MILD** (combined A− or A designation), and **SEV** (combined A+ or A+AD designation) for meaningful interpretation. Orthogonal contrasts of the least squared means to detect a linear relationship of severity categories with performance variables were reported and the orthogonal contrast of 0 vs. S, 0 vs. MILD, and 0 vs. SEV were considered.

The variable DUR was estimated by summing the number of data collections, per steer, where abnormal liver tissue was detected by ultrasound (proportion of 8 collections) and included as a continuous response variable when examining the fixed effects of treatment and liver severity. When determining the fixed effect of DUR on performance variables, however, detection of abnormal liver tissue was treated as count data with potential for up to eight abnormal detections. Counts were then grouped into categorical variables of length of DUR, where: **NEV** = only normal liver tissue detected at each ultrasound data collection, **SHORT** = steers with abnormal tissue detected at 1 or 2 data collections (estimated DUR of 28–56 d), **MED** = steers with abnormal tissue detected at 3 or 4 data collections (estimated DUR of 84–112 d), and **LONG** = steers with abnormal tissue detected at 5 or more ultrasound data collections (estimated >140 d). Orthogonal contrasts of the least squared means to detect a linear relationship of DUR and performance variables were reported and the orthogonal contrasts of NEV vs. SHORT, NEV vs. MED, and NEV vs. LONG were considered. Significance of all tests were established at *P* < 0.05 and tendencies determined as 0.05 < *P* ≤ 0.10.

## RESULTS AND DISCUSSION

### Treatment Effects

The effects of treatment on finishing cattle growth performance and carcass characteristics are presented in Tables 2 and 3, respectively. No differences (*P ≥* 0.155) were observed for mid-test BW, final BW, DMI, ADG, G:F or carcass adjusted performance between steers fed IGY or TYL. Hot carcass weight, dressing percentage, LMA, and MARB were similar between treatments (*P* ≥ 0.139), but steers consuming TYL had greater FT than steers consuming IGY (0.813 vs. 0.613 cm, respectively; *P =* 0.033). This resulted in TYL-fed steers having a greater calculated YG than IGY-fed steers (2.86 vs. 2.45, respectively; *P* = 0.020). The estimated EBF was also greater for TYL fed steers compared to IGY fed steers (27.5% vs. 26.3%, respectively: *P* = 0.042), but there was no treatment effect on AFBW (*P* = 0.169). Steers fed TYL resulted in a greater percentage of carcasses grading premium Choice than steers fed IGY (30.8% vs. 7.41%, respectively; *P* = 0.030), whereas steers fed IGY resulted in a greater percentage of carcasses grading low choice than steers fed TYL (59.3% vs. 30.8%, respectively; *P* = 0.038). While gain performance was not different between treatment groups, composition of gain may have been affected favoring greater fat deposition in TYL fed steers but at this time, it is not clear why this occurred. As this experiment was the first to examine this particular antibody combination, no literature exists to compare these findings between IGY and TYL. It was not anticipated that the egg yolk product would decrease the rate of fat deposition when compared to TYL, unless there was a difference in liver abscess rate, as liver abscesses have been shown to reduce 12th rib backfat thickness in beef carcasses (Brown and Lawrence, 2010). DiLorenzo et al. (2008) fed a similar IGY product containing both *Streptococcus bovis* and *F. necrophorum* antibody and did not detect a difference in gain performance or carcass characteristics when compared to control steers who were fed neither IGY nor TYL. In the current experiment, although DMI was not statistically different, the TYL fed steers consumed an average of 0.24 kg more per day than IGY fed steers which may account for the difference in fat composition at harvest.

**Table 2.** Effects of treatment on live and carcass adjusted feeding performance of Holstein steers

	Treatment^*^			
	IGY	TYL	SEM	*P*
Live performance				
Initial BW, kg	371	374	2.41	0.292
Mid-trial BW	519	515	4.28	0.455
Final BW, kg	643	647	7.94	0.703
DMI, kg				
d 0–84	9.52	9.82	0.147	0.155
d 85–188	9.38	9.45	0.112	0.780
d 0–188	9.45	9.69	0.146	0.233
ADG, kg				
d 0–84	1.77	1.67	0.064	0.278
d 85–188	1.19	1.28	0.090	0.476
d 0–188	1.45	1.45	0.038	0.947
G:F, kg				
d 0–84	0.188	0.172	0.008	0.173
d 85–188	0.124	0.134	0.009	0.397
d 0–188	0.153	0.149	0.003	0.419
Carcass-adjusted performance				
Final BW, kg^†^	642	647	8.75	0.694
ADG, kg^‡^	1.45	1.45	0.041	0.909
G:F, kg^§^	0.153	0.150	0.004	0.553

^*1^Treatments include: TYL = group fed tylosin phosphate (90 mh/hd/d) for entirety of trial and IGY = group fed immunoglobulin Y (2.5 g/hd/d) supplementation for entirety of trial.

^†^Values calculated by dividing HCW by the average DP of all steers on trial (61.8%).

^‡^Values calculated by subtracting carcass-adjusted final shrunk BW from the initial shrunk BW and divided by 188 d on feed.

^§^Values calculated as carcass adjusted ADG divided by the overall mean DMI.

**Table 3.** Effects of treatment on carcass characteristics and liver abscess severity distribution of Holstein steers

	Treatment^*^			
	IGY	TYL	SEM	*P*
HCW, kg	397	400	5.4	0.694
DP, %	61.8	61.9	0.33	0.875
FT, cm	0.613	0.813	0.0647	0.033
LMA, cm^2^	92.2	88.3	1.86	0.139
YG	2.45	2.86	0.123	0.020
MARB	434	443	18.3	0.716
Quality grade, %				
Prime	7.41	0.00	3.74	0.163
Premium choice	7.41	30.8	7.47	0.030
Low choice	59.3	30.8	9.53	0.038
Select	25.9	38.5	9.25	0.338
EBF, %^†^	26.3	27.5	0.43	0.042
AFBW, kg^†^	650	635	8.0	0.169
Liver abscess rate, %	48.2	65.4	9.8	0.213
Severity, %^‡^				
0	51.9	34.6	9.76	0.213
A−	14.8	26.9	8.02	0.286
A	3.70	0.00	2.69	0.331
A+	11.1	11.5	6.33	0.962
A+AD	18.5	26.9	8.33	0.475
DUR, %^§^	22.2	25.3	6.50	0.744

^*^Treatments include: TYL = group fed tylosin phosphate (90 mh/hd/d) for entirety of trial and IGY = group fed immunoglobulin Y (2.5 g/hd/d) supplementation for entirety of trial.

^†^Calculations for EBF and AFBW by (Guiroy et al., 2001) using the common EBF of 28% for cattle grading low choice.

^‡^Liver severity scores include: 0, no abscesses or scars; A−, one or two small abscess or resolved scar; A, one or two large abscesses or several small abscesses; A+, multiple large abscesses; A+AD, adhesion.

^§^The estimated duration of time that abnormal liver tissue was detected via ultrasound (proportion based on abnormal tissue detection at eight collections.)

Treatment did not affect the prevalence of abscessed livers, distribution of abscess severity, or the estimated DUR (*P* ≥ 0.213; Table 3). The current experiment is limited in interpretation of this outcome due to the lack of a negative control. However, DiLorenzo et al. (2008) reported liver abscess score on a continuous scale and found that steers fed a *F. necrophorum* antibody preparation had a 41.6% reduction in liver abscess severity score than control steers who were fed neither IGY nor TYL. The author attributed this reduction in liver abscess severity to lesser ruminal counts of *F. necrophorum*, which was made apparent in a previous experiment (DiLorenzo et al., 2006). The pool of literature for specifically formulated immunoglobulin-Y products developed for the control of liver abscesses in cattle is small (DiLorenzo et al., 2006, 2008). Research focused on other antigens, however, has been conducted in beef calves to control bovine rotavirus and bovine coronavirus using a similar oral route of administration resulting in significantly reduced viral shedding compared with calves not consuming the egg yolk product (Kuroki et al., 1994, 1997; Ikemori et al., 1997). More recent in vitro work by Zhen et al. (2008) and Xu et al. (2012) substantiated the concept of immunoglobulin-Y adherence to antigens as being dose-dependent when applied to *Fusobacterium* spp. or *Staphylococcus* spp. Outcomes of antigen selection, antibody combinations, and dosage, however, are yet to be established in the literature for the purposes of liver abscess control in cattle. Research determining optimal antibody combination and comparing customized IGY products with both negative controls (no feed additive) and positive controls (TYL) are needed before IGY can be considered as a suitable alternative to TYL in the cattle feeding industry. The outcomes of the current research add to the literature and may be used as an aid in determining an effect size for future experimental planning.

### Liver Abscess Severity Effects

No difference was detected in live performance or carcass-adjusted performance among liver abscess severity categories (*P* ≥ 0.262; Table 4); however, there was a tendency (*P* < 0.10) for a linear decrease (5.8%) in carcass-adjusted G:F as liver abscess severity increased. Failure to detect a difference in feeding performance between abscess severity categories was not unexpected due to a low number of experimental units for this type of assessment but the results are provided for the later discussion of abscess duration. Based on our parameters, the computed power for detecting a meaningful difference in final BW, DMI, ADG, G:F, and carcass-adjusted performance ranged from 5% to 42%, indicating much larger sample sizes were needed for determining the effects of abscess severity on performance variables. In a meta-analysis by Brink et al. (1990) where *n* = 566 beef steers, when comparing 0 and A+ liver scores, pens of cattle with a greater prevalence of liver abscesses (77%) resulted in a 4.8% reduction in DMI, a 13.5% reduction in carcass-adjusted ADG, and a 10.7% reduction in carcass-adjusted G:F.

**Table 4.** Effects of liver abscess severity on feeding performance and carcass characteristics of Holstein steers

	Liver abscess severity^1^							
	0	S	MILD	SEV			Contrasts^2^	
*n*=	23	6	6	18	SEM	*P*	*Lin*	*0 vs. Sev*
Live performance								
Final BW, kg	645	658	620	648	16.4	0.406	–	–
DMI, kg	9.50	9.59	9.48	9.68	0.312	0.895	–	–
ADG, kg	1.45	1.48	1.33	1.48	0.079	0.447	–	–
G:F, kg	0.152	0.154	0.140	0.153	0.007	0.399	–	–
Carcass-adjusted^3^								
Final BW, kg	651	660	629	638	18.2	0.516	–	–
ADG, kg	1.48	1.48	1.38	1.42	0.087	0.635	–	–
G:F,kg	0.156	0.155	0.145	0.147	0.0073	0.339	*	–
Carcass characteristics								
HCW, kg	402	408	389	394	11.2	0.516	–	–
DP, %	62.4^a^	62.0^a^	62.6^a^	60.8^b^	0.01	0.010	*	***
FT, cm	0.791	0.830	0.660	0.587	0.137	0.218	*	*
LMA, cm^2^	91.5	87.5	91.6	89.3	4.00	0.777	–	–
YG	2.70	2.98	2.46	2.54	0.269	0.450	–	–
MARB	454	438	438	419	38.4	0.700	–	–
EBF, %^4^	27.4	27.6	26.3	26.1	0.91	0.262	*	*
AFBW, kg^4^	640	644	637	647	17.3	0.929	–	–
DUR, %^5^	5.59^a^	16.7^a^	14.3^a^	52.4^b^	10.9	<0.0001	***	***

^1^Liver abscess severity: 0 = normal liver tissue, S = resolved abscess scar, MILD (combined liver scores of A− = one or two small abscess and A = one or two large abscesses or several small abscesses), and SEV (combined liver scores of A+ = multiple large abscesses, A+AD = adhesion).

^2^Linear orthogonal contrasts and non-orthogonal contrast of 0 vs. SEV subclass where *P* < 0.10*, P < 0.05**, and *P* < 0.005***.

^3^ Final BW values calculated by dividing HCW by the average DP (dressing percentage) of all steers on trial (61.8%). ADG calculated by subtracting carcass-adjusted final shrunk BW from the initial shrunk BW and divided by 188 d on feed and G:F calculated as carcass adjusted ADG divided by the overall mean DMI.

^4^Calculations for EBF and AFBW by (Guiroy et al., 2001) using the common EBF of 28% for cattle grading low choice.

^5^he estimated duration of time that abnormal liver tissue was detected via ultrasound (proportion based on abnormal tissue detection at eight collections.)

^a,b^Values are least squared means. Means without a common superscript differ at *P* < 0.05.

There was not enough power for detecting differences in carcass characteristics of HCW, LMA, YG, and MARB, or modeled AFBW between severity scores, therefore, those means are reported but not discussed (Table 4), but previous literature indicates lesser HCW, LMA, YG, and MARB among cattle with severe liver abscesses vs. normal (Brown et al., 1975; Rust et al., 1980; Brink et al., 1990; Brown and Lawrence, 2010). In the current experiment, power to detect differences in dressing percentage and FT were >99%. Carcasses with liver scores of 0, S, and MILD had greater DP than those with SEV scores (62.4%, 62.0%, 62.6% vs. 60.8%, respectively; *P* = 0.001) which is also consistent with previous research (Brown et al., 1975; Rust et al., 1980; Brink et al., 1990; Brown and Lawrence, 2010). It has been suggested that the lower DP of cattle exhibiting severely abscessed livers or those with abdominal adhesions is the result of greater carcass trimming (Brown and Lawrence, 2010) and greater visceral organ mass, as the liver compensates its mass when it becomes damaged. There was a tendency (*P* = 0.051) for a linear decrease in FT as liver abscess severity increased. Using two large datasets (total *n* > 75,000 steers and heifers), Brown and Lawrence (2010) also detected a linear decrease in FT as severity score increased. In the current experiment, there was a tendency for a linear decrease in EBF as liver abscess severity increased (*P* = 0.054). Reasons for decreased FT and EBF may include the diversion of metabolic energy toward greater heat production during liver mass compensation and lesser-retained energy for growth. No other studies have reported the effect of liver severity score on EBF or AFBW but these modeled variables, which account for variation between cattle types marketed for divergent quality grade endpoints, may be useful to include in future research, especially when using data from multiple experiments.

As expected, there was a significant relationship between liver abscess severity score and the estimated DUR (*P* < 0.001). Carcasses that had liver scores of 0, S, MILD, and SEV scores were estimated to have been affected by liver abnormalities for 5.59%, 16.7%, 14.3%, and 52.4% of the finishing period, respectively. It was not surprising to observe that steers with more severe liver abscesses would be burdened longer than those with S and MILD liver scores. It was surprising, however, to see that steers with normal livers at harvest were detected to have abnormalities during the feeding period. It is not known if the ultrasound detection of liver abnormalities within the normal liver group were simply “false positives” or potentially abscesses that occurred deeper in the liver tissue and resolved, as livers were not dissected and only surface scars were recorded. Future studies attempting liver ultrasound data collection may benefit from complete dissection of the post-mortem liver to verify the presence or absence of scar tissue beneath the liver surface.

### Liver Abscess Duration Effects

Although liver scores may be severe at harvest, the effect size of liver abscess severity on performance is likely also dictated by the duration of their burden, yet, there have been no investigations of abscess duration on feeding performance or carcass characteristics. The effects of DUR on finishing cattle growth performance and carcass characteristics are presented in Table 5, along with the distribution of severity scores within each of the grouped DUR categories, and the calculated power. Differences in final BW, DMI, ADG, or G:F among differing lengths of DUR were not found (*P* ≥ 0.119) likely due to lack of power. Power calculated from data used in the contrasts of NEV vs. LONG duration categories for effects on carcass-adjusted ADG and carcass-adjusted G:F were adequate for discussion (76.8% and 85.2%, respectively). Carcass-adjusted ADG was greater for steers classified as NEV vs. steers classified as LONG (1.49 vs. 1.30 kg, respectively; *P* = 0.025). The main effects of DUR on carcass-adjusted G:F revealed that only steers classified as LONG had reduced (11.1%) efficiency when compared to the mean of steers classified as NEV, SHORT, and MED (*P* = 0.044).

**Table 5.** Effects of estimated abscess duration on feeding performance and carcass characteristics of Holstein steers

	Estimated abscess duration^1^								
	NEV	SHORT	MED	LONG			Contrasts^2^		
*n*=	28	10	7	8	SEM	*P*	*Lin*	*0 vs. LONG*	Power, %^3^
Live performance									
Final BW, kg	648	634	659	633	15.2	0.463	–	–	31.7
DMI, kg	9.59	9.43	9.62	9.61	0.289	0.936	–	–	8.3
ADG, kg	1.46	1.38	1.57	1.38	0.072	0.170	–	–	67.7
G:F, kg	0.152	0.147	0.164	0.144	0.0062	0.119	–	–	75.3
Carcass-adjusted^4^									
Final BW, kg	654	637	650	619	16.5	0.239	–	*	41.3
ADG, kg	1.49	1.40	1.52	1.30	0.076	0.093	–	**	66.7
G:F,kg	0.155^a^	0.149^a^	0.158^a^	0.137^b^	0.0064	0.044	*	**	75.6
Carcass characteristics									
HCW, kg	404	394	402	383	10.2	0.239	–	*	39.7
DP, %	62.3^a^	62.1^ab^	60.9^bc^	60.5^c^	0.01	0.016	***	***	100
FT, cm	0.751	0.742	0.711	0.533	0.1299	0.465	–	–	29.8
LMA, cm2	89.3	94.9	85.5	89.1	3.25	0.186	–	–	51.7
YG	2.79	2.43	2.92	2.40	0.23	0.184	–	–	49.8
MARB	455	405	414	446	35.2	0.453	–	–	23.4
EBF, %^5^	27.4	26.1	27.0	25.9	0.84	0.233	–	*	33.1
AFBW, kg^5^	643	648	646	635	16.0	0.927	–	–	9.4
Liver severity distribution, count^6^									
0	21	3	2	0					
S	3	2	1	0					
MILD	4	1	0	1					
SEV	0	4	4	7					

^1^Categories of abnormal detections counts include: NEV = normal liver tissue detected at all ultrasound collections, SHORT = abnormal tissue detected at 1 or 2 collections (28–56 d), MED = abnormal tissue detected at 3 or 4 collections (84–112 d), and LONG = abnormal tissue detected at 5 or more collections (>140 d).

^2^Linear orthogonal contrasts and non-orthogonal contrast of NEV vs. LONG subclass where *P* < 0.10*, P < 0.05**, and *P* < 0.005***.

^3^ Power analysis conducted post hoc based on experimental results.

^4^Final BW values calculated by dividing HCW by the average dressing percentage (DP) of all steers on trial (61.8%). ADG calculated by subtracting carcass-adjusted final shrunk BW from the initial shrunk BW and divided by 188 d on feed and G:F calculated as carcass adjusted ADG divided by the overall mean DMI.

^5^Calculations for EBF and AFBW by (Guiroy et al., 2001) using the common EBF of 28% for cattle grading low choice.

^6^Distribution of the varied liver scores within the duration classifications. 0 = normal liver tissue, S = resolved abscess scar, MILD (combined liver scores of A− = one or two small abscess and A = one or two large abscesses or several small abscesses), and SEV (combined liver scores of A+ = multiple large abscesses, A+AD = adhesion).

^a,b,c^Values are least squared means. Means without a common superscript differ at *P* <0.05.

The experimental design was not powerful enough to detect differences in HCW, FT, LMA, YG, MARB, EBF, or AFBW between steers exhibiting different abscess duration estimates (*P* ≥ 0.184; Table 5). Dressing percentage, however, was linearly affected by abscess duration where steers in the NEV group had the greatest dressing % (62.3%) and was different from steers in MED and LONG duration groups (60.9% and 60.5%, respectively). The severity distribution within the MED and LONG duration categories were two normal livers, one scarred liver, one mildly abscessed and eleven severely abscessed. These results suggest that steers affected for 84 days or more by liver abscesses may have reduced dressing percentage, regardless of abscess severity at harvest, when compared to steers in which abscesses were never detected by ultrasound. Interestingly, 8 of the 15 steers exhibiting severe abscess scores at harvest were classified as SHORT or MED in duration, and their carcass-adjusted G:F was not different than steers classified as NEV (0.149, 0.158, and 0.155 kg, respectively) but was greater than steers classified as LONG (0.144 kg). This evidence suggests that severity score at harvest is not the only explanatory variable which may affect feeding performance and carcass characteristics. As the distribution of abscess severity scores among different DUR categories illustrates, duration of liver abscess burden during the finishing phase varies even among steers with common severity scores. Differences in DUR may account for some variability when examining the effects of abscess severity on performance. While a laborious endeavor, estimating the duration of abscess burden during the finishing period may further our knowledge on the efficacy of in-feed products used for liver abscess control. It may be that reducing abscess duration is just as important as reducing abscess severity.

## CONCLUSION

In this experiment, we did not have evidence of a treatment effect on the prevalence of abscessed livers, abscess severity, and either live or carcass-adjusted performance. Steers fed TYL had greater EBF and a greater proportion of carcasses that graded premium choice, than did steers fed IGY. Further research will be needed to evaluate the use of IGY as a replacement for TYL. Regarding abscess severity impact on performance among Holstein steers, our results concur with previous research derived from beef-type steers that with increasing severity of liver abscess there was tendency for some performance measurements (carcass-adjusted G:F, dressing %, FT, and EBF) to linearly decrease. Abscess severity, however, only partially explains these effects. We provide evidence that the duration of abscess burden during the finishing period may also be responsible for losses in performance and carcass value, even among carcasses with similar liver severity scores.

## References

[CIT0001] Amachawadi R. G., and Nagaraja T. G. 2016 Liver abscesses in cattle: a review of incidence in Holsteins and of bacteriology and vaccine approaches to control in feedlot cattle. J. Anim. Sci. 94(4):1620–1632. doi:10.2527/jas.2015-0261 27136021

[CIT0002] Beukers A. G., Zaheer R., Cook S. R., Stanford K., Chaves A. V., Ward M. P., and McAllister T.. 2015 Effect of in-feed administration and withdrawal of tylosin phosphate on antibiotic resistance in enterocci isolated from feedlot steers. Front. Microbiol 6:483. doi:10.3389/fmicb.2015.00483 PMC4444845 26074889

[CIT0003] Braun U 2009 Ultrasonography of the liver in cattle. Vet. Clin. North Am. Food Anim. Pract. 25:591–609, Table of Contents. doi:10.1016/j.cvfa.2009.07.003 19825435

[CIT0004] Brink D. R., Lowry S. R., Stock R. A., and Parrott J. C.. 1990 Severity of liver abscesses and efficiency of feed utilization of feedlot cattle. J. Anim. Sci. 68:1201–1207. doi:10.2527/1990.6851201x 2365638

[CIT0005] Brown H., Bing R. F., Grueter H. P., McAskill J. W., Cooley C. O., and Rathmacher R. P.. 1975 Tylosin and Chlortetracycline for the prevention of liver abscesses, improved weight gains, and feed efficiency in feedlot cattle. J. Anim. Sci 40:207–213. doi:10.2527/jas1975.402207x 1116959

[CIT0006] Brown T. R., and Lawrence T. E.. 2010 Association of liver abnormalities with carcass grading performance and value. J. Anim. Sci. 88:4037–4043. doi:10.2527/jas.2010-3219 20817855

[CIT0007] Depenbusch B. E., Drouillard J. S., Loe E. R., Higgins J. J., Corrigan M. E., and Quinn M. J.. 2008 Efficacy of monensin and tylosin in finishing diets based on steam-flaked corn with and without corn wet distillers grains with solubles. J. Anim. Sci. 89(9):2270–2276. doi:10.2527/jas.2007-0017 18469059

[CIT0008] DiLorenzo N., Dahlen C. R., Diez-Gonzales F., Lamb G. C., Larson J. E., and DiCostanzo A.. 2008 Effects of feeding polymicrobial antibody preparations on rumen fermentation patterns, performance, and carcass characteristics of feedlot steers. J. Anim. Sci. 86:3023–3032. doi:10.2527/jas.2008-0859 PMC7110015 18955541

[CIT0035] DiLorenzo N., Diez-Gonzalez F., and DiCostanzo A.. 2006 Effects of feeding polyclonal antibody preparations on ruminal bacterial populations and ruminal pH of steers fed high-grain diets. J. Anim. Sci. 84:2178–2185. doi:10.2527/jas.2005-489 16864880 PMC7109817

[CIT0009] Eastwood L. C., Boykin C. A., Harris M. K., Arnold A. N., Hale D. S., Kerth C. R., Griffin D. B., Savell J. W., Belk K. E., Woerner D. R., et al. 2017 National Beef Quality Audit-2016: transportation, mobility, and harvest-floor assessments of targeted characteristics that affect quality and value of cattle, carcasses, and by-products1. Trans. Anim. Sci 1:229–238. 10.2527/tas2017.0029 PMC7250433 32704647

[CIT0010] Eisemann J. H., Huntington G. B., and Catherman D. R.. 1996 Patterns of nutrient interchange and oxygen use among portal-drained viscera, liver, and hindquarters of beef steers from 235 to 525 kg body weight. J. Anim. Sci. 74:1812–1831. doi:10.2527/1996.7481812x 8856436

[CIT0011] Guiroy P. J., Fox D. G., Tedeschi L. O., Baker M. J., and Cravey M. D.. 2001 Predicting individual feed requirements of cattle fed in groups. J. Anim. Sci. 79:1983–1995. doi:10.2527/2001.7981983x 11518207

[CIT0012] Huntington G. B., and Reynolds C. K.. 1987 Oxygen consumption and metabolite flux of bovine portal-drained viscera and liver. J. Nutr. 117:1167–1173. doi:10.1093/jn/117.6.1167 3298581

[CIT0013] Ikemori Y., Ohta M., Umeda K., Icatlo F. C. Jr., Kuroki M., Yokoyama H., and Kodama Y.. 1997 Passive protection of neonatal calves against bovine coronavirus-induced diarrhea by administration of egg yolk or colostrum antibody powder. Vet. microbial., 58(2–4):105–111. doi:10.1016/s0378-1135(97)00144-2 PMC7117124 9453122

[CIT0014] Kuroki M., Ohta M., Ikemori Y., Icatlo F. C. Jr, Kobayashi C., Yokoyama H., and Kodama Y.. 1997 Field evaluation of chicken egg yolk immunoglobulins specific for bovine rotavirus in neonatal calves. Arch. Virol. 142:843–851. doi:10.1007/s007050050123 9170509

[CIT0015] Kuroki M., Ohta M., Ikemori Y., Peralta R. C., Yokoyama H., and Kodama Y.. 1994 Passive protection against bovinerotavirus in calves by specific immunoglobulins from chicken egg yolk. Arch. Virol., 138:143–148. doi:10.1007/BF01310045 7980004

[CIT0016] Jacob M. E., Fox J. T., Narayanan S. K., Drouillard J. S., Renter D. G., and Nagaraja T. G.. 2008 Effects of feeding wet corn distillers grains with solubles with or without monensin and tylosin on the prevalence and antimicrobial susceptibilities of fecal foodborne pathogenic and commensal bacteria in feedlot cattle. J. Anim. Sci. 86:1182–1190. doi:10.2527/jas.2007-0091 18192558

[CIT0018] Larsson A., and Sjöquist J.. 1990 Chicken IgY: utilizing the evolutionary difference. Comp. Immunol. Microbiol. Infect. Dis. 13:199–201. doi:10.1016/0147-9571(90)90088-b 2076606

[CIT0019] Lechtenberg K. F., and Nagaraja T. G.. 1991 Hepatic ultrasonography and blood changes in steers with experimentally induced liver abscesses. Am. J. Vet. Res 52(6):803−809. 1679304

[CIT0020] Lechtenberg K. F., Nagaraja T. G., Leipold H. W., and Chengappa M. M.. 1988 Bacteriologic and histologic studies of hepatic abscesses in cattle. Am. J. Vet. Res. 49:58–62. 3354968

[CIT0021] McKeith R. O., Gray G. D., Hale D. S., Kerth C. R., Griffin D. B., Savell J. W., Raines C. R., Belk K. E., Woerner D. R., Tatum J. D., et al. 2012 National Beef Quality Audit-2011: harvest-floor assessments of targeted characteristics that affect quality and value of cattle, carcasses, and byproducts’. J. Anim. Sci. 90(13):5135–5142. doi:10.2527/jas.2012-5477 22952370

[CIT0022] Nagaraja T. G., and Chengappa M. M.. 1998 Liver abscesses in feedlot cattle: a review. J. Anim. Sci. 76:287–298. doi:10.2527/1998.761287x 9464910

[CIT0023] Nagaraja T. G., and Lechtenberg K. F.. 2007 Liver abscesses in feedlot cattle. Vet. Clin. North Am. Food Anim. Pract. 23:351–69, ix. doi:10.1016/j.cvfa.2007.05.002 17606156

[CIT0034] National Academies of Sciences, Engineering, and Medicine 2016 Nutrient requirements of beef cattle. National Academies Press.

[CIT0024] Polanowski A., Zabłocka A., Sosnowska A., Janusz M., and Trziszka T.. 2012 Immunomodulatory activity accompanying chicken egg yolk immunoglobulin Y. Poult. Sci. 91:3091–3096. doi:10.3382/ps.2012-02546 23155018 PMC7195453

[CIT0025] Reinhardt C. D., and Hubbert M. E.. 2015 Control of liver abscesses in feedlot cattle: A review. Prof. Am. Sci 31:101–108. doi: 10.15232/pas.2014-01364

[CIT0026] Rust S. R., Owens F. N., and Gill D. R.. 1980 Liver abscesses and feedlot performance. Oklahoma Cattle Feeder’s Day Rep. MP 107 Accessed at: https://pdfs.semanticscholar.org/ddea/0a8857215e3addabb07831f892a855cb9f25.pdf. Accessed August 2020.

[CIT0028] Tan Z. L., Nagaraja T. G., and Chengappa M. M.. 1996 Fusobacterium necrophorum: Virulence factors, pathogenic mechanism and control measures. Vet. Res. Commun. 20(2):113–140. doi:10.1007/BF00385634 8711893

[CIT0029] USDA 2017 United States standards for grades of carcass beef-§54.105 Effective date December 18, 2017 Washington, DC: Agricultural Marketing Service, USDA https://www.ams.usda.gov/sites/default/files/media/CarcassBeefStandard.pdf. Accessed August 2020.

[CIT0030] USDA 2019 Antimicrobial Use and Stewardship on U.S. Feedlots, 2017 Fort Collins, CO: USDA-APHIS-VS-NAMS. May, 2019.

[CIT0033] Xu F.X., Xu Y.P., Jin L.J., Liu H., Wang L.H., You J.S., Li S.Y., and Li X.Y.. 2012 Effectiveness of egg yolk immunoglobulin (IgY) against periodontal disease-causing Fusobacterium nucleatum. J. Appl. Microbiol. 113:983–991. doi:10.1111/j.1365-2672.2012.05396.x. 22789022

[CIT0031] Zaheer R., Cook S. R., Klima C. L., Stanford K., Alexander T., Topp E., Read R. R., and McAllister T. A.. 2013 Effect of subtherapeutic vs. therapeutic administration of macrolides on antimicrobial resistance in Mannheimia haemolytica and enterococci isolated from beef cattle. Front. Microbiol. 4:133. doi:10.3389/fmicb.2013.00133 23750157 PMC3664329

[CIT0032] Zhen Y.-H., Jin L.-J., Guo J., Li X.-Y., Lu Y.-N., Chen J., and Xu Y.-P.. 2008 Characterization of specific egg yolk immunoglobulin (IgY) against mastitis-causing Escherichia coli. Vet. Microbiol. 130:126–133. doi:10.1016/j.vetmic.2007.12.014. 18255238

